# A workshop on asthma management programs and centers in Brazil: reviewing and explaining concepts*

**DOI:** 10.1590/S1806-37132015000100002

**Published:** 2015

**Authors:** Rafael Stelmach, Alcindo Cerci Neto, Ana Cristina de Carvalho Fernandez Fonseca, Eduardo Vieira Ponte, Gerardo Alves, Ildely Niedia Araujo-Costa, Laura Maria de Lima Belizário Facury Lasmar, Luci Keiko Kuromoto de Castro, Maria Lucia Medeiros Lenz, Paulo Silva, Alberto Cukier, Alexssandra Maia Alves, Aline Silva Lima-Matos, Amanda da Rocha Oliveira Cardoso, Ana Luisa Godoy Fernandes, Bruno Piassi de São-José, Carlos Antônio Riedi, Deborah Schor, Décio Medeiros Peixoto, Diego Djones Brandenburg, Elineide Gomes dos Santos Camillo, Faradiba Sarquis Serpa, Heli Vieira Brandão, João Antonio Bonfadini Lima, Jorge Eduardo Pio, Jussara Fiterman, Maria de Fátima Anderson, Maria do Socorro de Lucena Cardoso, Marcelo Tadday Rodrigues, Marilyn Nilda Esther Urrutia Pereira, Marti Antila, Sonia Maria Martins, Vanessa Gonzaga Tavares Guimarães, Yara Arruda Marques Mello, Wenderson Clay Correia de Andrade, William Salibe-Filho, Zelina Maria da Rocha Caldeira, Álvaro Augusto Souza da Cruz-Filho, Paulo Camargos

**Affiliations:** University of São Paulo, School of Medicine, Hospital das Clínicas, São Paulo, Brazil. Department of Pulmonology, Heart Institute, Hospital das Clínicas, Faculdade de Medicina da Universidade de São Paulo - HC-FMUSP, University of São Paulo School of Medicine Hospital das Clínicas - São Paulo, Brazil; State University at Londrina, Londrina, Brazil. (Paraná) State University at Londrina; and Coordinator. Programa Respira Londrina (Breathe, Londrina Program), Londrina, Brazil; Programa Criança que Chia, Belo Horizonte, Brazil. Programa Criança que Chia (Wheezing Child Program), Belo Horizonte City Hall, Belo Horizonte, Brazil; Jundiaí School of Medicine, Jundiaí, Brazil. Jundiaí School of Medicine, Jundiaí, Brazil; Fortaleza Municipal Department of Health, Fortaleza, Brazil. Programa de Atenção Integral à Criança e Adulto com Asma de Fortaleza - PROAICA, Integrated Asthma Management Program for Chidren and Adults in Fortaleza - Fortaleza Municipal Department of Health, Fortaleza, Brazil; University Hospital, Federal University of Maranhão, São Luís, Brazil. Asthma Patient Care Program, Federal University of Maranhão University Hospital, São Luís, Brazil; Federal University of Minas Gerais, Belo Horizonte, Brazil. Federal University of Minas Gerais; and Pediatric Pulmonologist. Centro Multidisciplinar para Asma de Difícil Controle - CEMAD, Multidisciplinary Center for the Treatment of Difficult-to-Control Asthma - and Programa Criança que Chia (Wheezing Child Program), Belo Horizonte City Hall, Belo Horizonte, Brazil; Programa Respira Londrina, Londrina, Brazil. Programa Respira Londrina (Breathe, Londrina Program), Londrina, Brazil; Conceição Hospital Group, Porto Alegre, Brazil. Asthma Program, Conceição Hospital Group, Porto Alegre, Brazil; Asthma Patient Management Program, Montenegro, Brazil. Asthma Patient Management Program - RESPIRAÇÃO - Montenegro, Brazil; University of São Paulo, School of Medicine, Hospital das Clínicas, São Paulo, Brazil. Department of Pulmonology. Heart Institute, University of São Paulo School of Medicine Hospital das Clínicas, São Paulo, Brazil; Fortaleza Municipal Department of Health, Fortaleza, Brazil. Fortaleza Municipal Department of Health, Fortaleza, Brazil; Bahia State Asthma Control Program, Salvador, Brazil. Programa para Controle da Asma na Bahia - ProAR, Bahia State Asthma Control Program - Salvador, Brazil; Goiânia Municipal Department of Health, Goiânia, Brazil. Programa Catavento (Pinwheel Program), Goiânia Municipal Department of Health, Goiânia, Brazil; Federal University of São Paulo, Paulista School of Medicine, Department of Pulmonology, São Paulo, Brazil. Department of Pulmonology, Federal University of São Paulo Paulista School of Medicine, São Paulo, Brazil; Federal University of Minas Gerais, Hospital das Clínicas, Belo Horizonte, Brazil. Pulmonology Outpatient Clinic, Federal University of Minas Gerais Hospital das Clínicas, Belo Horizonte, Brazil; Federal University of Paraná, Curitiba, Brazil. Federal University of Paraná, Curitiba, Brazil; Federal University of Pernambuco, Hospital das Clínicas, Recife, Brazil. Allergy Outpatient Clinic, Recife Allergology Center; and Volunteer Preceptor. Asthma Outpatient Clinic, Federal University of Pernambuco Hospital das Clínicas, Recife, Brazil; Federal University of Pernambuco, Mother and Child Department, Recife, Brazil. Mother and Child Department, Federal University of Pernambuco, Recife, Brazil; Porto Alegre Hospital de Clínicas, Montenegro, Brazil. Porto Alegre Hospital de Clínicas, Porto Alegre, Brazil and Asthma Patient Management Program - RESPIRAÇÃO - Montenegro, Brazil; Conceição Hospital Group, Porto Alegre, Brazil. Conceição Hospital Group, Porto Alegre, Brazil; School of Medical Sciences, Santa Casa de Misericórdia de Vitória, Vitória, Brazil. Asthma Program, Santa Casa de Misericórdia de Vitória School of Medical Sciences, Vitória, Brazil; Bahia State University at Feira de Santana, Department of Pediatrics, Feira de Santana, Brazil. Department of Pediatrics, Bahia State University at Feira de Santana; and Coordinator. Feira de Santana Asthma and Allergic Rhinitis Control Program, Feira de Santana, Brazil; Porto Alegre Municipal Department of Health, Porto Alegre, Brazil. Asthma Program, Porto Alegre Municipal Department of Health, Porto Alegre, Brazil; Rio de Janeiro Municipal Department of Health, Rio de Janeiro, Brazil. Rio de Janeiro Municipal Department of Health, Rio de Janeiro, Brazil; Pontifical Catholic University of Rio Grande do Sul, School of Medicine, Porto Alegre, Brazil. Pontifícia Universidade Católica do Rio Grande do Sul - PUCRS, Pontifical Catholic University of Rio Grande do Sul - School of Medicine, Porto Alegre, Brazil; Brazilian Association of Asthma Patients, Rio de Janeiro, Brazil. Associação Brasileira de Asmáticos - ABRA, Brazilian Association of Asthma Patients - Rio de Janeiro, Brazil; Federal University of Amazonas, Manaus, Brazil. Programa de Assistência e Controle da Asma - PACA, Asthma Care and Control Program - and Associate Professor. Universidade Federal do Amazonas - UFAM, Federal University of Amazonas - Manaus, Brazil; Santa Casa Sisters of Mercy, Hospital of Porto Alegre, Porto Alegre, Brazil. Universidade de Santa Cruz do Sul - UNISC, University of Santa Cruz do Sul - Santa Cruz do Sul, Brazil; and Pulmonologist. Pereira Filho Ward, Irmandade da Santa Casa de Misericórdia de Porto Alegre - ISCMPA, Santa Casa Sisters of Mercy Hospital of Porto Alegre - Porto Alegre, Brazil; Uruguaiana Municipal Department of Health, Uruguaiana, Brazil. Programa Infantil de Prevenção de Asma - PIPA, Children's Asthma Prevention Program - Uruguaiana Municipal Department of Health, Uruguaiana, Brazil; Sorocaba Municipal Asthma Program, Sorocaba, Brazil. Sorocaba Municipal Asthma Program, Sorocaba, Brazil. Physician. Programa Respira Rio, Rio de Janeiro, Brazil. Programa Respira Rio (Breathe, Rio Program), Rio de Janeiro, Brazil; Brazilian Society of Family and Community Medicine, Rio de Janeiro, Brazil. Grupo de Trabalho de Problemas Respiratórios - GRESP, Working Group on Respiratory Problems - Sociedade Brasileira de Medicina de Família e Comunidade - SBMFC, Brazilian Society of Family and Community Medicine - Rio de Janeiro, Brazil; Brasília Mother and Child Hospital, Brasília, Brazil. Programa de Atendimento ao Paciente Asmático do Distrito Federal - PAPA-DF, Asthma Patient Management Program in the Federal District of Brasília - and Supervisor. Residency Program in Pediatric Allergy and Immunology, Brasília Mother and Child Hospital, Brasília, Brazil; Associação Brasileira de Asmáticos, São Paulo, Brazil. Department of Allergy and Immunology, Edmundo Vasconcelos Hospital Complex; and Director. Associação Brasileira de Asmáticos-São Paulo - ABRA, Brazilian Association of Asthma Patients-SP - São Paulo, Brazil; Itabira Municipal Department of Health, Itabira, Brazil. Projeto Respirai (Breathe Project), Itabira Municipal Department of Health, Itabira, Brazil; São Camilo University Center, School of Medicine, São Paulo, Brazil. Department of Pulmonology, ABC School of Medicine, Santo André, Brazil; and Professor. São Camilo University Center School of Medicine, São Paulo, Brazil; Associação Brasileira de Asmáticos, São Paulo, Brazil. Niterói Municipal Health Foundation, Niterói, Brazil. Pediatrician and Director of Government Policy and International Relations. Associação Brasileira de Asmáticos-São Paulo - ABRA, Brazilian Association of Asthma Patients-SP - São Paulo, Brazil; Federal University of Bahia, School of Medicine, Salvador, Brazil. Federal University of Bahia School of Medicine; and Coordinator. Center of Excellence in Asthma, Salvador, Brazil; Federal University of Minas Gerais, Department of Pediatrics, Belo Horizonte, Brazil. Department of Pediatrics, Federal University of Minas Gerais, Belo Horizonte, Brazil

**Keywords:** Asthma, Academic medical centers, Area health education centers, Health planning organizations, Regional medical programs, Managed care programs

## Abstract

**Objective:**

To report the results of a workshop regarding asthma management programs and centers (AMPCs) in Brazil, so that they can be used as a tool for the improvement and advancement of current and future AMPCs.

**Methods:**

The workshop consisted of five presentations and the corresponding group discussions. The working groups discussed the following themes: implementation of asthma management strategies; human resources needed for AMPCs; financial resources needed for AMPCs; and operational maintenance of AMPCs.

**Results:**

The workshop involved 39 participants, from all regions of the country, representing associations of asthma patients (n = 3), universities (n = 7), and AMPCs (n = 29). We found a direct relationship between a lack of planning and the failure of AMPCs. Based on the experiences reported during the workshop, the common assumptions about AMPCs in Brazil were the importance of raising awareness of managers; greater community participation; interdependence between primary care and specialized care; awareness of regionalization; and use of medications available in the public health system.

**Conclusions:**

Brazil already has a core of experience in the area of asthma management programs. The implementation of strategies for the management of chronic respiratory disease and their incorporation into health care system protocols would seem to be a natural progression. However, there is minimal experience in this area. Joint efforts by individuals with expertise in AMPCs could promote the implementation of asthma management strategies, thus speeding the creation of treatment networks, which might have a multiplier effect, precluding the need for isolated centers to start from zero.

## Abstract (pt)

**Objetivo:**

Relatar os resultados de uma oficina de trabalho sobre programas e centros de atenção a asmáticos (PCAAs) no Brasil para que possam servir como instrumento para melhoria e avanço dos PCAAs existentes e criação de novos.

**Métodos:**

A oficina de trabalho constituiu-se de cinco apresentações e discussões em grupos. Os grupos de trabalho discutiram os seguintes temas: implementação de uma linha de cuidado em asma; recursos humanos necessários para os PCAA; recursos necessários para financiar os PCAA; e manutenção do funcionamento dos PCAAs.

**Resultados:**

A oficina envolveu 39 participantes de todas as regiões do país, representando associações de asmáticos (n = 3), centros universitários (n = 7) e PCAAs (n = 29). Evidenciou-se uma relação direta entre a ausência de planejamento e o insucesso dos PCAAs. Com base nas experiências brasileiras elencadas durante a oficina, as premissas comuns foram a importância da sensibilização do gestor, maior participação da comunidade, interdependência entre a atenção primária e a especializada, observação da regionalização e utilização dos medicamentos disponíveis no sistema público de saúde.

**Conclusões:**

O Brasil já tem um núcleo de experiências na área programática da asma. A implementação de uma linha de cuidado em doenças respiratórias crônicas e sua inclusão nas redes de saúde parecem ser o caminho natural. Porém, a experiência nessa área ainda é pequena. Agregar pessoas com experiência nos PCAAs na elaboração da linha de cuidado em asma encurtaria tempo na criação de redes de atenção com possível efeito multiplicador, evitando que se partisse do zero em cada local isolado.

## Introduction

Some asthma patient management programs and centers that are currently in operation in Brazil are coming of age. In an editorial, Holanda^(^ 1 ^)^ reported results of questionnaires on Asthma Management Programs and Centers (AMPCs) in Brazil, completed by 16 members of the Brazilian Thoracic Association (BTA) or regional affiliates. At the time, 14 AMPCs (87.5%) confirmed that they were in regular operation, 10 of which had been established in the 1990s. Looking back, the responses regarding asthma management seem alarming: inhaled medications were unavailable (there were only oral medications); treatment demand was higher than treatment availability; and there were no outpatient clinics specializing in asthma.

Many AMPCs were created as a result of the dissemination of the first national and international guidelines for the management of asthma, published in the same decade. Therefore, those guidelines prompted the holding of the First and Second Brazilian Conferences on Asthma in 1997 and 1999, respectively. The unavailability of inhaled corticosteroids (ICs) in public institutions contradicted the cornerstone of the treatment of persistent asthma, derived from the guidelines. Pioneers of that time include the AMPCs in the cities of Belo Horizonte, Fortaleza, and São Paulo, which already showed that educating patients decreased the number of hospitalizations and improved patient quality of life.^(^ 1 ^,^ 2 ^)^

One group of authors^(^ 2 ^)^ prepared a timeline of the evolution of public policies and AMPCs in Brazil since 1996, showing that, in 1998, the National Drug Policy was created, which led to the dispensation of medications for asthma control. This provision stimulated and gave support to the creation of new programs and required the implementation of referral centers.^(^ 2 ^)^ It is not by chance that the *Carta de Salvador* (Salvador Charter),^(^ 3 ^)^ urging the implementation of the National Asthma Control Program, was formulated in 2001. The cities of Porto Alegre, Goiânia, Londrina, Niterói, Salvador, Feira de Santana, Rio de Janeiro, and Vitória established their AMPCs at the same time as decrees regulating the allocation of federal resources to health care services were being issued.^(^ 2 ^)^ At that time, in addition to ensuring care for asthma patients, some AMPCs stood out for their scientific production, others stood out for providing training to professionals from different areas, and others stood out for expanding to small municipalities, decentralizing activities.

In 2007, an editorial^(^ 4 ^)^ presented an account of the decade and highlighted the need for professional training and funding to advance the quality of care by improving AMPCs. Examples of asthma programs in Brazil were obtained in 2008, by analyzing the responses to the forms sent to BTA members and members of the Brazilian Association of Allergists and Immunologists.^(^ 5 ^)^ Of 55 services that reported having a systematized program, 11 (20%) did not respond to the structural questions and 27 (49%) were excluded from the analysis (17 treated severe asthma and 10 had had the program for less than two years). All 17 programs analyzed received public funding for their maintenance: 4 (23%) received state funding exclusively, whereas 13 (77%) received state and municipal funding. There were no programs in northern Brazil. All 17 programs had referral centers with specialists, and 47% developed educational activities (lectures or individual visits) within the community. In addition, 47% provided home visits by nurses, and 41% adopted public health strategies, such as family health care, outreach, humanizing practices, and visits by community health care agents. That study^(^ 5 ^)^ showed that, from 2003 onward, the number of programs increased significantly, as a result of the availability of full public funding for the purchase of asthma medications.

Some successful programs no longer exist because of political and administrative changes. However, it can be stated that there has been no dissemination of programs across the country. There are still fewer than five dozen programs, as mentioned above. Most are supported by the dedication of some individuals-experts in their field-and often with resources from funding agencies, partnerships with the private sector, or both. In the majority of localities, they have not become programs or management strategies of the municipal or state departments of health, especially since, as yet, the Brazilian National Ministry of Health itself has not prioritized management strategies for chronic respiratory diseases.

Between 1991 and 2010, the epidemiologic picture changed, as a result of the growth of the Brazilian population at a rate of 20 million per decade. The population jumped from 146.8 million in 1991 to 190.7 million in 2010.^(^ 6 ^)^ This means that there was an increase in the number of asthma patients. Conversely, the number of hospitalizations for asthma via the *Sistema Único de Saúde* (SUS, Brazilian Unified Health Care System) decreased from 400,000 per year to fewer than 200,000 per year between 2000 and 2012,^(^ 7 ^)^ with a disproportionate decrease of 30% in the expenditures for such hospitalizations (110 million Brazilian reals vs. 80 million Brazilian reals). It is correct to state that the asthma centers and programs helped this reduction in the number of hospitalizations. In addition, they encouraged the changes made by the laws and national regulations that initially led to the decentralization of payment of costs of asthma and rhinitis medications, as well as to the publication of the Primary Care Guidebook - rhinitis , asthma, and COPD^(^ 8 ^)^ and, more recently, of the revised Clinical Protocol and Therapeutic Guidelines - asthma.^(^ 9 ^)^ Because of the high prevalence of COPD, which is a mandatory differential diagnosis for asthma, COPD care was combined with asthma care in adults in some centers, in addition to being standardized at the federal level.^(^ 10 ^)^ Given the regulation of the basic (i.e. municipal) and specialized (i.e. state) components of pharmaceutical care, as well as the free provision of basic asthma medications at enrolled pharmacies since 2012, it can be stated that there already is adequate public funding.^(^ 2 ^)^ However, the population's demands for more resources/materials for the treatment of chronic respiratory diseases have become a reality for public and private health, including via litigation.^(^ 11 ^)^ Another change in public health has been the increased value placed on the family health program strategy. According to the Brazilian National Ministry of Health,^(^ 12 ^)^ half of the Brazilian population receive some care through that strategy, and one of the current developments is the controversial program of importation of physicians. Conversely, several national and international studies have shown that asthma is not controlled in more than 50% of the patients evaluated,^(^ 13 ^)^ which can be confirmed by the still low use of ICs.^(^ 14 ^)^

What to do to provide care for those who remain unassisted and to continue to decrease asthma-related morbidity and mortality in Brazil is the question. The Global Initiative for Asthma (GINA)^(^ 15 ^)^ recommends that, by 2015, there should be fewer than 100,000 hospitalizations per year for asthma in Brazil. It must be borne is mind that, despite the decrease in the number of hospitalizations, official statistics show that the number of deaths from asthma (3,000 deaths per year) has remained unchanged.^(^ 7 ^)^ How to continue the history of AMPCs, making them a reference in education and care, is the challenge ahead. How to multiply them (physically or conceptually) is a challenge and a requirement. In an attempt to answer these questions, it was proposed that a workshop on AMPCs in Brazil be held. The objective of the present study was to report the results of this workshop so that they can be used as a tool for the improvement and advancement of current centers and programs, as well as for the establishment of new ones.

## Methods

Using a list of AMPCs that were identified in surveys conducted in 2000, 2008, and 2013 (the last survey has not been published), four coordinators of AMPCs that have been in operation since the 1990s selected health professionals with related activities throughout Brazil. The geographic distribution, lifetime (continuity), and infrastructure of AMPCs were taken into consideration, as were their scientific production, training of staff specializing in chronic respiratory diseases, and successful or innovative experiences in the area. Some professionals involved in tertiary care (severe asthma), as well as individuals who were in charge of associations of asthma patients and had health training, were also selected. In addition, physicians, nurses, and pharmacists who were directly involved in the processes in AMPCs and who preferably did not perform program management were invited. Two workshop coordinators made a list of 48 guests after analysis of AMPCs, as well as of associations of patients in the states of Rio de Janeiro and São Paulo.

The objectives proposed for the workshop were as follows:

- Compile successful experiences in AMPCs in Brazil and the difficulties in implementing asthma management strategies in the SUS.
- Outline the current state of initiatives in asthma in Brazil in their various phases (planning and regional integration; professional training and standardization; funding and management; expansion and consolidation; and national guidelines for asthma programs).
- Find and propose solutions to problems associated with the development of the AMPCs that are already in operation in the country.
- Develop a practical manual for the implementation of program activities and centers of excellence for the treatment of asthma.

**Chart 1 -:**
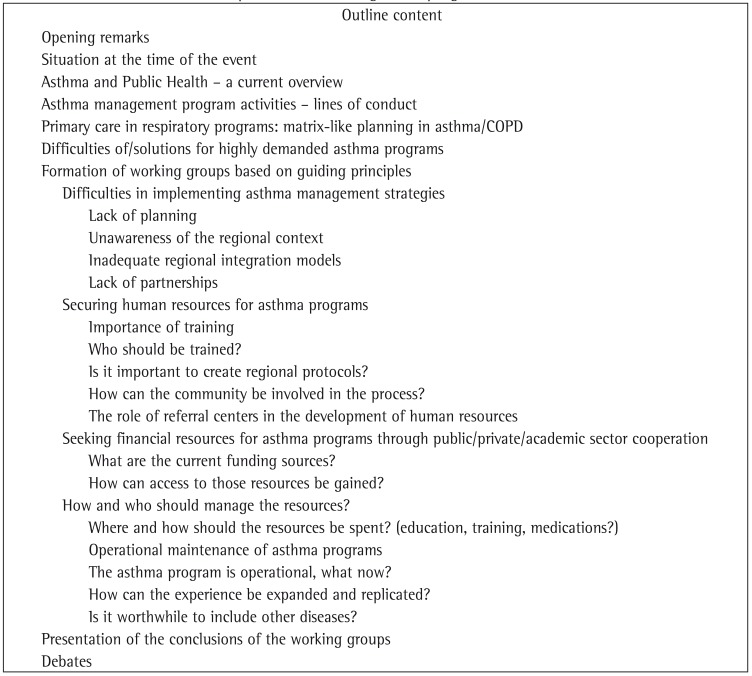
Outline of the workshop on asthma management programs and centers.

The workshop agenda was designed to favor group work. The coordinators defined the proposed themes for discussion. Presentations on specific issues served as a basis for the group discussions. Each group had two coordinators, who were in charge of recording the discussions and reporting them at plenary sessions. Chart 1 presents a summary of the workshop program.

The group discussions were recorded in real time by a company specializing in editing for events. The results were systematized by combining the group reports, the notes made by the (workshop and group) coordinators, and the text prepared by the editing company. The results are presented by group discussion topic.

## Results

Of 48 guests, 39 attended the event, which was held in the city of São Paulo and lasted eight hours. All regions of the country were represented (the Northern, Central West, Northeastern, Southern, and Southeastern regions had 1, 2, 8, 11, and 17 representatives, respectively). The group of professionals consisted of 13 pulmonologists, 8 pediatric pulmonologists, 6 allergists/immunologists, 6 pediatricians (2 of whom were allergologists), 2 family/community physicians, 3 pharmacists, and 1 nurse. The associations of asthma patients had 3 representatives present. Seven professionals represented university (secondary/tertiary) referral centers, and 29 represented AMPCs. Although three of those centers/AMPCs had private management, all were directed to SUS.

The results of the study groups were systematized and are described as follows:

## Difficulties in implementing asthma management strategies

Lack of planning

- Strategic planning is critical in implementing asthma management strategies, in order to minimize potential implementation difficulties and maximize community and public manager awareness.
- Through planning, public managers are committed to political and financial support, even when there are local political changes.
- In order to maintain the support from public managers, asthma management strategies should have an appropriate cost-benefit ratio and prioritize the free provision of outpatient asthma medications, because this reduces hospitalization expenditures and increases the productivity of patients and health professionals.
- It is important to estimate the number of patients who will be reached by the intervention. Management strategies that prioritize primary care help a large volume of patients who, individually, do not use the health care system very often. When management strategies prioritize secondary care, they will help a smaller volume of patients who individually use many health resources. In the long run, no level of care should be excluded, and there should be mechanisms to allow patients to seek treatment networks or webs according to the behavior of their disease.
- Planning focused on the development of continuous data collection tools provides indicators that can be used to measure the benefits and impact of those activities in public health.
- Civil society and medical societies must work together to avoid setbacks in the treatment of asthma patients, and, therefore, public awareness should be raised.
- Medical specialty societies and patient associations have a very positive record in fostering the implementation of asthma management strategies.
- Currently, asthma programs are organized around individuals, and there is a high risk of loss of continuity.

Regional context unawareness /inadequate regional integration models

- Asthma management strategies should respect the heterogeneity of the country. There is already a minimal health care system that reaches almost the entire country-primary care clinics (PCCs) and family health program teams/strategies-which should be prioritized.
- Asthma management strategies should take advantage of the structure that is available. Specialized centers are indispensable to training primary care teams without experience in asthma and to supporting patients who are refractory to treatment, while also making that structure available to smaller municipalities (in the region).

Lack of partnerships

- In order to implement and maintain asthma management strategies, it is necessary to have support from the public/state sector, the private sector (i.e., schools, pharmaceutical industries, the media, and health insurance plans), and the third sector (i.e., universities, religious institutions, nongovernmental organizations, foundations, associations, etc.).
- The physical structure, the purchase of medications, and the health teams are the responsibility of municipalities and states. The private sector and the third sector can contribute by disseminating information, providing technological knowledge, and facilitating public/state sector activities. These contributions can mainly take the form of scientific meetings for health teams, donation of spirometers, support for forming associations of asthma patients, media dissemination of information about asthma to the lay public, and commitment for selling medications to the public sector at the lowest price possible.
- The use of volunteers should be encouraged. Presentations in schools and communities given by volunteers have significant impact, according to experiences in the city of São Paulo. It is of note that volunteer activities are not regular and should be planned as short-term.

## Securing human resources for AMPCs

Importance of training

- Continuing training of all categories of health professionals at all levels of care is necessary so that professionals working in asthma care know how to identify, classify, and manage patients appropriately, thereby reducing asthma-related morbidity and mortality, as well as improving the quality of life of patients and their family members.
- In addition to health professionals, it is of paramount importance to train patients and their family members in recognizing the disease, the periods of exacerbation, and the forms of treatment, therefore preventing complications.
- Currently, the focus of public health is the Family Health Program Strategy, which is responsible for the holistic care of individuals. The objective is to train primary care professionals so that they can identify people with asthma, classify them in accordance with the clinical protocols, and manage them, in order to reduce the number of unnecessary referrals to secondary care and not to place an additional burden on the health system.
- In addition to training, it is important that the network be under continuing supervision for the maintenance of appropriate care, with qualified referrals, well-defined network flows, and monitoring of local indicators.

Who should be trained?

- Training should be attended by the multidisciplinary team together as a group, and a piece of information that is shared by all categories should be used in demonstrating the role of each individual in patient care. Techniques such as matrix-like planning can make the function of each professional in daily practice clearer. Any trained professional can be a local tutor.
- Professionals at referral centers should also be trained to receive, accommodate, and treat patients in accordance with specialized care protocols.
- The creation of regional centers could enable training and consulting anywhere in the country through partnerships, preventing extensive traveling within the country. Professionals from societies, institutions, and universities who are associated with the asthma problem in our communities can be part of those centers.

Is it important to create regional protocols?

• With regard to the creation of regional protocols, it is of note that the current national and international patient care guidelines are applicable to all centers. Indeed, It is necessary to create flows and organize health care services according to local contexts.

How can the community be involved in the process?

- A community that is aware of the risks and costs of illness, and that knows that there are resources to reduce them, mobilizes with public authorities to try to improve care for users.
- Community involvement should be broad, considering individuals with or without asthma, but with an emphasis on the asthma community, with includes patients and their family members.
- Knowledge about asthma and asthma management strategies should be disseminated widely, and this can be made possible through local health councils and through local/regional media (television, radios, churches, and schools). The dissemination of information through widely accessed electronic media such as social networks reaches large populations.
- Emphasize to patients that they are responsible for demanding complete, quality care, which includes professionals who provide appropriate care, provision of necessary materials, and continuing education. This responsibility cannot be assumed only by health professionals.
- Children should start receiving asthma education early, which, in addition to strengthening family ties in terms of education, encourages adherence to treatment.

The role of referral centers in the development of human resources

- During training, referral centers are responsible for passing on information regarding patient referral flow and asthma management strategy steps to the entire system. This knowledge should be shared by all professionals working in the system, from primary care professionals to professionals working in referral centers, whether they are regional/secondary or tertiary centers.
- Referral centers are also responsible for providing information to primary care professionals and for, together with those professionals, defining criteria for patient referral to/from referral centers and criteria for patient follow-up. Standardization of patient follow-up and management, as well as of referral and counter-referral models, facilitates dialogue.

## Seeking financial resources for asthma programs through public/private/academic sector cooperation

What are the current funding sources?

- From a broad perspective, funding is partially taken care of, especially when it comes to medications. The lack of funding implies discontinuity of activities and loss of motivation.
- The federal government makes asthma medications available through the special drugs program and the "Popular Pharmacy" program. Some municipalities contribute by making ICs and short-acting bronchodilators available at PCCs.
- There is a need for an alignment between what is proposed in the guidelines/strategies and what in fact there is to treat patients and control asthma.

How can access to those resources be gained?

- The group discussions evidenced the need for the current forms of funding and for alternatives to be further explained in a document prepared by public health specialists with a deep understanding of the subject. This document would be produced by specialist societies or organizations/associations related to asthma.
- There are forms and sources of funding that are unknown to the group.
- The provision of special medications, with the addition of long-acting β_2_ agonists, was an advance; however, in some programs, this provision is not far reaching, because it is focused on people (it is not standardized).

How and who should manage the resources?

- Social control is very important because it fosters the continuity of the program.
- The raising and allocation of resources should be based on technical rather than on political criteria.
- A joint management strategy with the family health program should be pursued. Since primary care is a priority and resources are directed to it, it is not feasible to have resources available for the asthma program unless there is integrated patient care. There is no money specifically for asthma, but there is money for primary care.
- In addition, there are funding sources other than the federal government, such as state and municipal foundations, through which it is possible to obtain additional resources.

Where and how should the resources be spent?

- It was concluded that there is no conflict between the recommendations in national and international guidelines and the medications provided to patients. Medications should be addressed in all stages of planning guidelines in the program.
- Funding for asthma education is as necessary as is funding for other activities. The resource exists, but it is imperative to understand and propose ways to apply for it.

## Operational maintenance of asthma programs

The present work group chose to merge the three questions that should be included in the program-"The asthma program is operational, what now?"; "How can the experience be expanded and replicated?"; and "Is it worthwhile to include other diseases?-into a single theme, which is described below.

Strategies for maintaining, expanding, and replicating asthma programs

- AMPCs are characterized as a set of pre-defined activities, objectives, and goals that will meet the needs of a population.
- The maintenance of AMPCs requires the following: knowledge of the local context; multidisciplinary coordination and institutional programs; greater awareness and continuing updating of professionals; dissemination of activities; and participation of the population.
- It was identified that comprehensiveness of care is important in individual care; however, when it comes to programs, maintaining the focus on care for people with asthma is critical. There should be integration with programs targeted at other conditions, such as smoking and COPD. For instance, smoking family members of children with asthma should be advised and referred to smoking cessation groups. The management of allergic rhinitis, however, should be addressed in asthma clinical protocols, given the prevalent association between rhinitis and asthma.
- Knowledge of the local context, together with definition of the area of operation and delimitation of the target population, favors the maintenance of the process. Cultural issues should be part of that knowledge and should guide activities that respect diversity.
- Program coordination should be multidisciplinary (pharmacists, family physicians, specialists, physical therapists, etc.), which provides different perspectives on the program and facilitates informed planning decisions based on the local context.
- Personal ownership-"So-and-so's program"-should be avoided. The program, whenever possible, must have a name of its own and institutional guidelines.
- Epidemiological data, such as prevalence and impact of asthma, should be used to raise awareness of and update professionals, being part of the educational process.
- Protocols or guidelines should be adapted for local use on the basis of current strategies, such as those of GINA and of the BTA. These protocols should include the different resources, whether structural or human, of primary care (PCCs), secondary care (specialty outpatient clinics), or tertiary care (emergency rooms and hospitals), with well-defined referral criteria and with an emphasis on treatment networks.
- The activities of the program and the assessment indicators should be disseminated to the population, managers, and directly involved professionals, as well as to the academic community (through conferences, symposia and publications). The information should be clear and objective. It is considered important to include communication techniques in the training of professionals. These individuals and groups can pass on technical information to the population, both individually and collectively, in health care clinics, local health councils, schools, associations of asthma patients, etc. The dissemination can be achieved through electronic means, newsletters, and more objective reports.
- It is important to provide managers with updated local epidemiological data and results for cost reduction and improvement in the quality of life of patients and their family members, as well as data regarding the involvement of the population.

## Discussion

The results of the present study confirm the importance of planning. We found a direct relationship between a lack of planning and the failure of asthma programs in their various phases (design, implementation, and maintenance). In the experiences reported during the workshop, there were shared assumptions in the planning phase of AMPCs in Brazil: greater awareness of managers; greater community participation; interdependence between primary care and specialized care; awareness of the regional context; and use of medications available in the public health system for the treatment of asthma. This is consistent with the medical literature,^(^ 16 ^,^ 17 ^)^ but there are some differences in the hierarchy of these assumptions.

The literature shows that one of the essential conditions for the implementation and maintenance of asthma programs is previous planning. In 2012, one group of authors^(^ 16 ^)^ highlighted the importance of and the need for the creation of a planning group that, from the beginning, involves all segments that will play a role in the asthma programs, including managers. This critical step of the process should be guided by knowledge of possible difficulties of the health care system, which are accessed through established indices.^(^ 16 ^,^ 18 ^)^

The GINA guidelines for improving care in asthma^(^ 16 ^)^ and the recommendations published by the BTA^(^ 17 ^)^ are currently two of the major sources of technical information and methods for implementation of asthma programs. However, hearing the players themselves in their work processes, which was made possible by the present study, revealed a scenario is that differs from those guidelines/recommendations in some aspects and complies with them in others. The workshop format in the present study allowed a very productive exchange of information among the various initiatives in Brazil and contributes to making up for the lack of scientific publications.

The results of the present study made it clear that the population is the major player in the process, because, in addition to being the object of the intervention, it is the major element that should initiate and monitor the program implementation process. Various studies have shown that interventions in the community, in order to provide greater educational and scientific support, lead to improved results.^(^ 19 ^)^ Social participation does not consist only of a monitoring role, but also of joint and multidisciplinary initiatives, such as dissemination of social knowledge.

It is also clear from the results that awareness of and decision making by public managers are determining factors for the success or failure of program activities. This is a characteristic of Brazilian society and is not related directly to health, but rather to politics,^(^ 20 ^)^ especially since the asthma interventions in the community that are mentioned in the present study^(^ 5 ^)^ were designed in state or municipal departments of health or in public universities. It is essential to understand that the role of the programs is both technical and political, which requires knowledge of legislation, system organization, public health strategies, etc.^(^ 17 ^)^ The issue of participation of managers permeates continuity solutions that can directly affect program maintenance. The involvement of collegiate bodies or the conversion of programs into state/municipal laws or decrees, such as the newly launched *Programa Respira Minas* (Breathe, Minas Program),^(^ 21 ^)^ reduces the possibility of discontinuity.

The understanding that the various levels of care should work jointly and in a coordinated manner was reinforced. The notion of interdisciplinary and multidisciplinary teams assuming a role in management strategies is internationally well known, and the singularity of professional categories is recognized, using these unique characteristics in order to improve team efficacy.^(^ 18 ^,^ 22 ^,^ 23 ^)^

Referral centers are support centers for patients who are more severely ill or for those who require technological or therapeutic resources. In addition, they can be responsible for training activities and for continuing and permanent education. In the models in Brazil, referral centers are also centers of program planning and management. Currently, managers invest most health resources in primary care, which can cause imbalance in management strategies. The balance between the different levels of care is an essential condition for any management strategy.^(^ 17 ^)^ According to the results of the present study, in centers where there are no specialists, it is necessary that specialists be available regionally.

Another important component in the implementation of AMPCs is the process of education through training of all health professionals^(^ 24 ^)^ and of patients themselves. One group of authors showed that the implementation of education programs leads to a reduction in asthma attacks, decreasing the number of hospitalizations and emergency room visits, as well as bringing about improvement in the quality of life of asthma patients.^(^ 25 ^)^

Continuing multidisciplinary training of primary care professionals has been shown to have a direct relationship with fewer asthma-related hospitalizations and better-informed referrals to specialties^(^ 19 ^-^ 22 ^)^ The vast majority of primary care professionals in Canada preferred training based on a combination of didactic lecture and clinical case discussion.^(^ 26 ^)^ More rational prescription of medications, well-structured action plans, and the proper use of spirometry led to improved care for asthma patients. In addition, according to the participants in the present study, professional training should address management strategies, especially referral and counter-referral criteria and flows.

The Clinical Protocol and Therapeutic Guidelines^(^ 9 ^)^ available in the SUS includes almost all therapeutic classes for asthma treatment, but their indications are for different situations than those recognized by national and international guidelines.^(^ 15 ^,^ 27 ^)^ Nevertheless, the workshop reiterated that it is not necessary to create new clinical protocols (also known as guidelines for asthma), but only to adapt them to local contexts and medical contexts.

There are resources for the purchase of medications (municipal resources) and for medical specialties (mostly state resources).^(^ 28 ^,^ 29 ^)^ The lack of resources-or the unawareness of their availability-is often cited as a limiting factor for the development of program implementation, but there are various activities that can be developed without financial resources.^(^ 16 ^)^

The involvement of other professionals and the transformation of programs into institutional policies rather than people-centered policies are key factors for success. Success has already been achieved in asthma programs in the cities of Belo Horizonte and Salvador,^(^ 30 ^-^ 33 ^)^ where there are several publications reporting national and international indicators of quality.

It was concluded that, even without appropriate dissemination, Brazil already has a core of experience in the area of asthma management programs, through local and regional activities, as well as activities in universities. Despite the fact that the movement for the creation of asthma programs has contributed to the current design of funding for treatment of the disease and has certainly influenced the epidemiological change regarding the disease, there has been no proliferation of AMPCs. Since 2003, the number of AMPCs in operation has remained virtually unchanged. Although this is not a phenomenon occurring just in Brazil,^(^ 16 ^)^ the national experience of AMPCs is sufficiently mature and has a critical mass of experienced professionals to come up with proposals^(^ 5 ^)^ for change.

The implementation of national strategies for the management of respiratory diseases and their incorporation into health care system protocols would seem to be a natural progression. However, there is minimal experience in management strategies in this area. Joint efforts by individuals, such as the present workshop participants, with expertise in AMPCs and availability to go to interested centers, who could act as facilitators to developing standards and methods and who could raise awareness of managers, would speed the creation of treatment networks and have a multiplier effect, thus precluding the need for isolated centers to start from zero.
